# Communication of Lung Cancer Screening Results: A National Survey of Veterans Health Administration Facilities

**DOI:** 10.1016/j.chpulm.2025.100183

**Published:** 2025-05-24

**Authors:** Elena Vlachos, John Dell’Italia, Kristina Crothers, Nicholas Maurice, Nichole T. Tanner

**Affiliations:** a Department of Pulmonary and Critical Care Medicine, Medical University of South Carolina, Charleston, SC; b Ralph H. Johnson VA Healthcare System, Charleston, SC; c Birmingham VA Healthcare System, Birmingham, AL; d Puget Sound VA Healthcare System, Seattle, WA; e Department of Pulmonary, Critical Care, Emory University, Atlanta, GA; f Atlanta VA Healthcare System, Atlanta, GA

To the Editor:

A key component to high-quality lung cancer screening (LCS) includes structured reporting of results.^1^ Methods of results delivery to patients vary and although studies indicate satisfaction with letters, these may lead to discordant understanding of results.^2^, ^3^, ^4^

Beginning in 2020, the Veterans Health Administration (VA) launched the Lung Precision Oncology Program network that includes > 107 LCS hub and spoke sites.^5^ The Lung Precision Oncology Program LCS Communication of Findings workgroup sought to characterize current practices for communication of results to inform best practices.

## Methods

**Figure 1:**
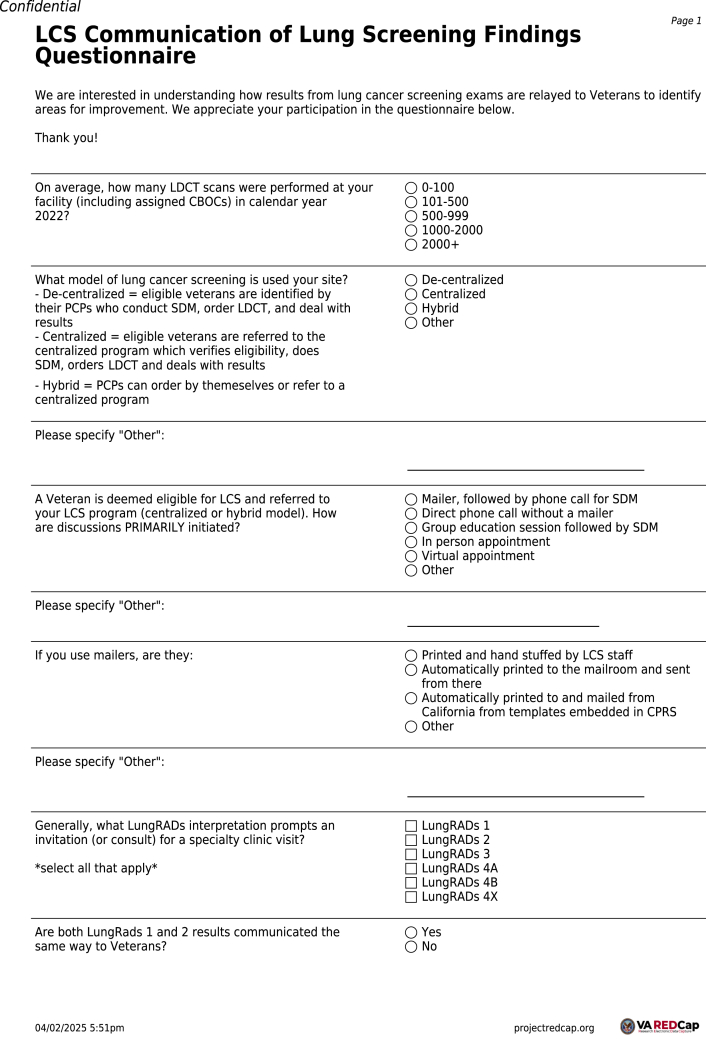

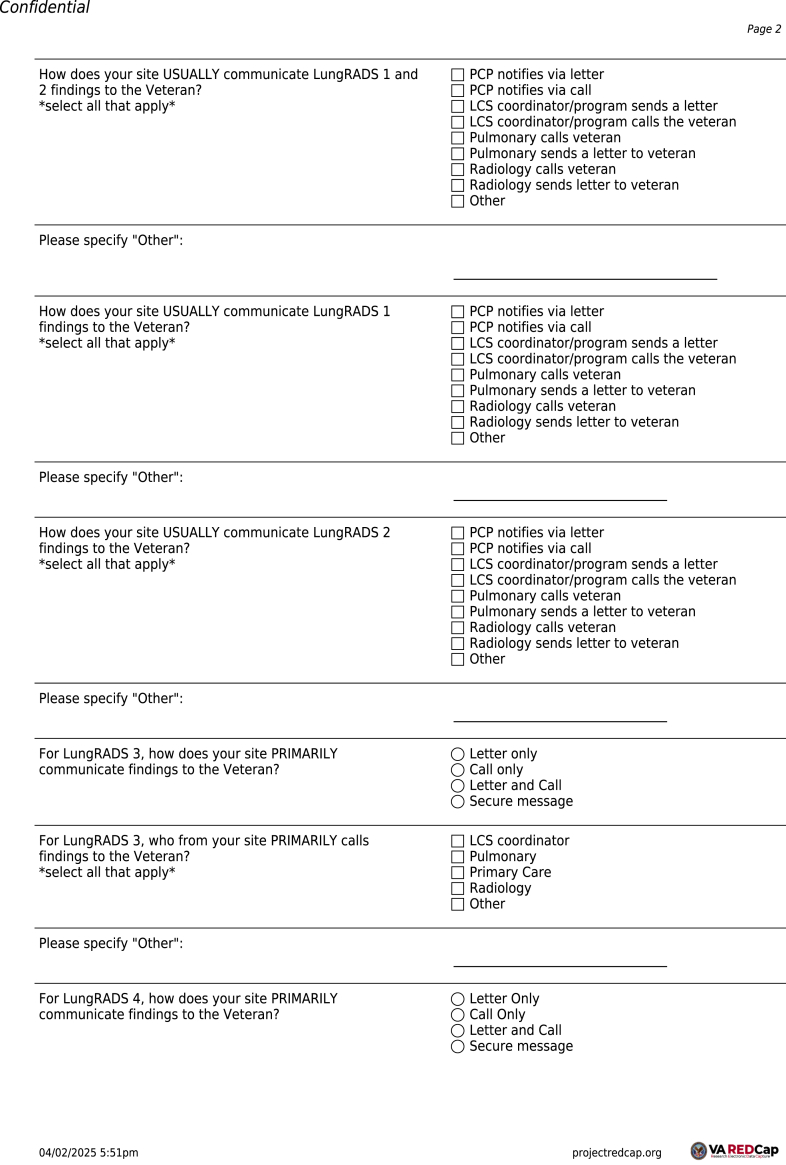

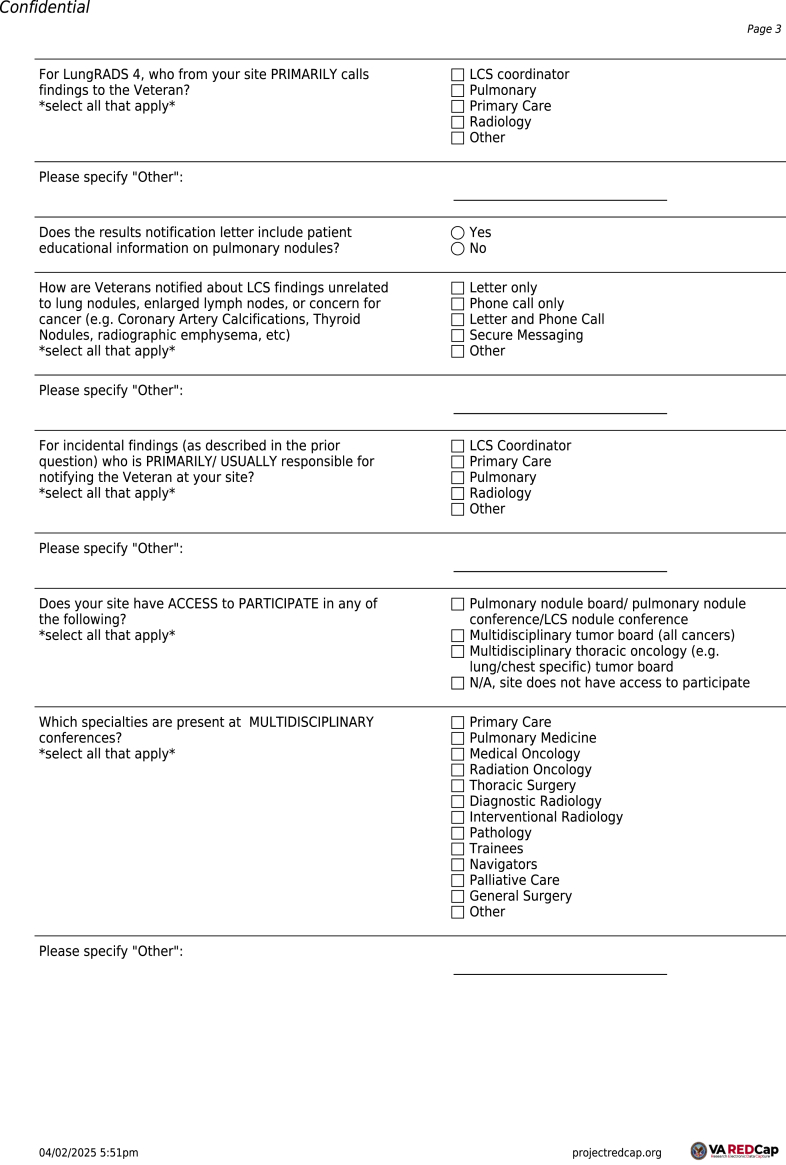

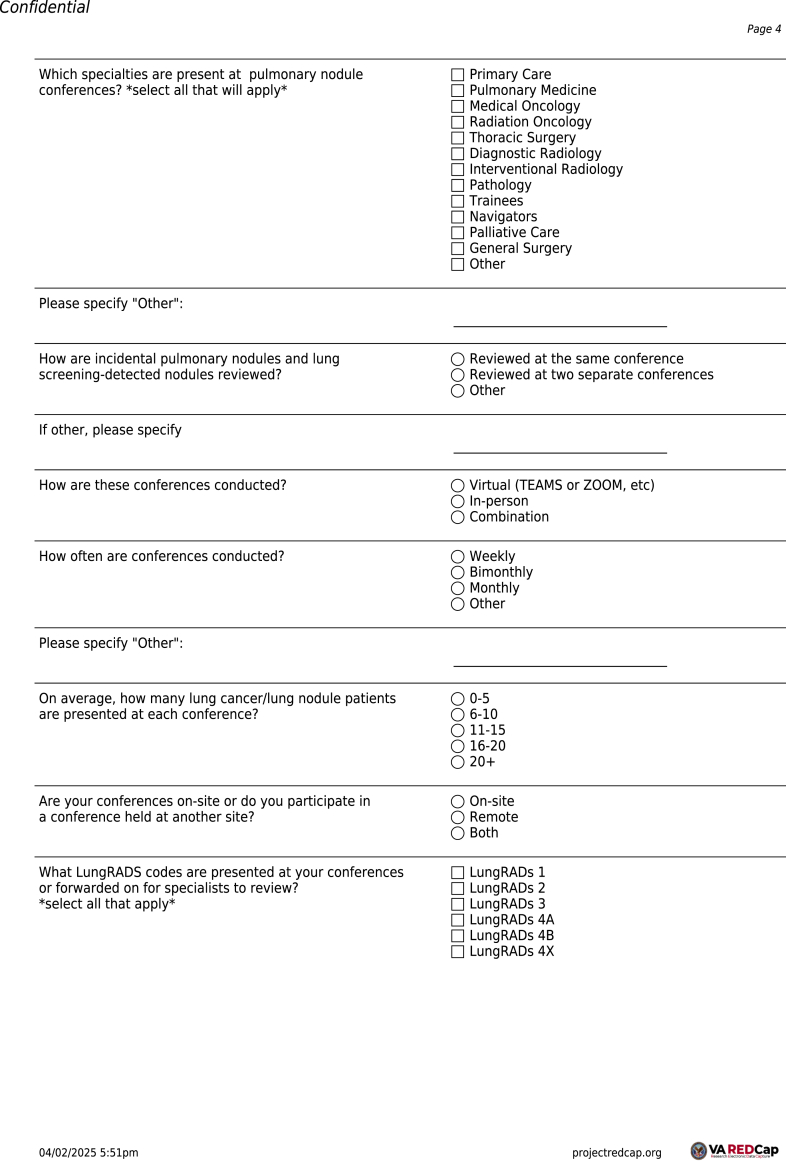

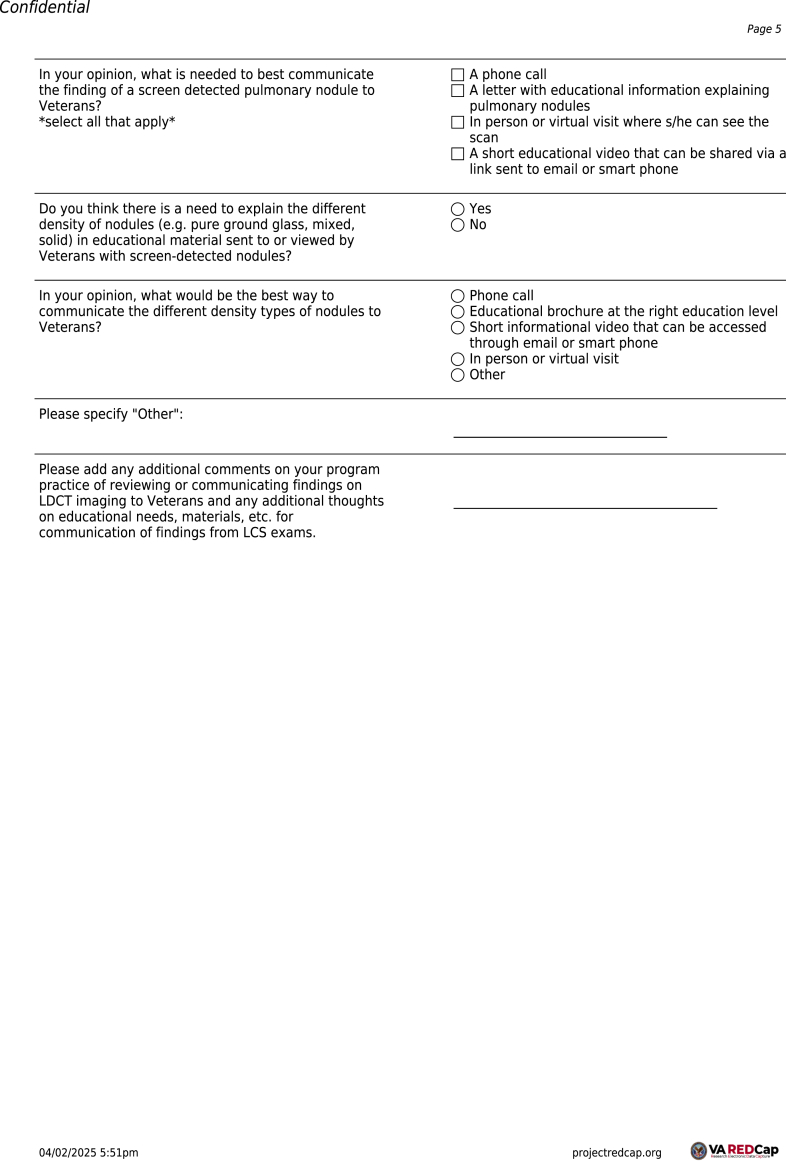
Survey questions and answer options. LDCT = low-dose CT; CBOC = community-based outpatient clinic; CPRS = computerized patient record system; N/A = not appliable; PCP = primary care provider; SDM = shared decision-making.

This cross-sectional descriptive survey was designed by LCS workgroup members, who evaluated and iteratively revised on content, format, and face validity. VA LCS coordinators anonymously completed the 29-item survey between January and March 2023. Up to 3 emails were sent to invite survey participants. Survey topics included LCS screening models, shared decision-making (SDM), modes of reporting screening results to patients based on LungRADs score, and management of other significant incidental findings (SIFs) (Fig 1). The institutional review board of the Medical University of South Carolina approved this as a non-research activity. Results were analyzed using simple descriptive statistics between July and August 2023.

## Results

Overall, 49 of 63 invited VA facilities (78%) responded to the survey. A total of 63 of 91 LCS coordinators (69%) completed the survey. LCS program models included centralized (48%), hybrid (44%), and decentralized (8%). The volume of low-dose CT (LDCT) scans performed in 2022 was > 2,000 in one-third of responding sites (Table 1). Elements of the SDM process include a previously developed paper shared decision aid^6^ and SDM visit to further discuss risks, benefits, and preferences for LCS. Participants indicated that SDM was conducted by phone call (53%). One-half of participants who conduct SDM via telephone (24%) indicated that the shared decision aid was also provided in the mail before the phone call. A minority indicated that SDM was conducted during in-person appointments (16%).

**Table 1:** Lung Cancer Screening Coordinator Survey Responses

Question	No. (%)
On average, how many LDCT scans were performed at your facility (including assigned CBOCs) in calendar year 2022? (n = 63)	
0-100	6 (9.5)
101-500	10 (15.9)
500-999	8 (12.7)
1000-2000	18 (28.6)
≥ 2000	21 (33.3)
What model of lung cancer screening is used your site? (n = 63)	
Decentralized	5 (7.9)
Centralized	24 (38.1)
Hybrid	27 (42.9)
Other	7 (11.1)
A veteran is deemed eligible for LCS and referred to your LCS program (centralized or hybrid model). How are SDM discussions primarily initiated? (n = 51)	
Mailer, followed by phone call for SDM	12 (23.5)
Direct phone call without a mailer	15 (29.5)
Group education session followed by SDM	0 (0)
In-person appointment	8 (15.7)
Virtual appointment	4 (7.8)
Other	12 (23.5)
Generally, what LungRADs interpretation prompts an invitation (or consult) for a specialty clinic visit? (n = 63)	
LungRADs 1	1 (1.6)
LungRADs 2	1 (1.6)
LungRADs 3	12 (19)
LungRADs 4A	46 (73)
LungRADs 4B	59 (93.7)
LungRADs 4X	59 (93.7)
Are both LungRads 1 and 2 results communicated the same way to veterans? (n = 63)	
Yes	56 (88.9)
No	7 (11.1)
How does your site usually communicate LungRADS 1 findings to the veteran? (n = 7)	
PCP notifies via letter	5 (71.5)
PCP notifies via call	2 (28.6)
LCS coordinator/program sends a letter	4 (57.2)
LCS coordinator/program calls the veteran	0 (0)
Pulmonary calls veteran	0 (0)
Pulmonary sends a letter to veteran	1 (14.3)
Radiology calls veteran	0 (0)
Radiology sends letter to veteran	0 (0)
Other	1 (14.3)
How does your site usually communicate LungRADS 2 findings to the veteran? (n = 7)	
PCP notifies via letter	4 (57.2)
PCP notifies via call	2 (28.6)
LCS coordinator/program sends a letter	4 (57.2)
LCS coordinator/program calls the veteran	3 (42.9)
Pulmonary calls veteran	1 (14.3)
Pulmonary sends a letter to veteran	1 (14.3)
Radiology calls veteran	0 (0)
Radiology sends letter to veteran	0 (0)
Other	1 (14.3)
How does your site usually communicate LungRADS 1 and 2 findings to the veteran? (n = 56)	
PCP notifies via letter	14 (25)
PCP notifies via call	10 (18)
LCS coordinator/program sends a letter	43 (76.8)
LCS coordinator/program calls the veteran	17 (30.4)
Pulmonary calls veteran	3 (5.4)
Pulmonary sends a letter to veteran	1 (1.8)
Radiology calls veteran	0 (0)
Radiology sends letter to veteran	0 (0)
Other	1 (1.8)
For LungRADS 3, how does your site primarily communicate findings to the veteran? (n = 63)	
Letter only	13 (20.6)
Call only	12 (19)
Letter and call	38 (60.4)
Secure message	0 (0)
For LungRADS 3, who from your site primarily calls findings to the veteran? (n = 61)	
LCS coordinator	37 (58.7)
Pulmonary	7 (11.1)
Primary care	14 (22.2)
Radiology	0 (0)
Other	3 (4.8)
For LungRADS 4, how does your site primarily communicate findings to the veteran? (n = 63)	
Letter only	0 (0)
Call only	28 (44.4)
Letter and call	35 (55.6)
Secure message	0 (0)
For LungRADS 4, who from your site primarily calls findings to the veteran? (n = 63)	
LCS coordinator	42 (66.7)
Pulmonary	19 (30.2)
Primary care	19 (30.2)
Radiology	1 (1.6)
Other	8 (12.7)
Does the results notification letter include patient educational information on pulmonary nodules? (n = 35)	
Yes	26 (74.3)
No	9 (25.7)
In your opinion, what is needed to best communicate the finding of a screen-detected pulmonary nodule to veterans?^a^ (n = 63)	
Phone call	51 (81)
Letter with educational information explaining pulmonary nodules	46 (73)
In-person or virtual visit where she/he can see the scan	21 (33.6)
Short educational video that can be shared via a link sent to email or smart phone	10 (16)
Do you think there is a need to explain the different density of nodules (eg, pure ground glass, mixed, solid) in educational material sent to or viewed by veterans with screen-detected nodules? (n = 63)	
Yes	23 (36.5)
No	40 (63.5)
In your opinion, what would be the best way to communicate the different density types of nodules to veterans? (n = 23)	
Phone call	3 (13.0)
Educational brochure at the right education level	12 (52.2)
Short informational video that can be accessed through email or smart phone	2 (8.7)
In-person or virtual visit	1 (4.3)
Other	5 (2.2)
How are veterans notified about LCS findings unrelated to lung nodules, enlarged lymph nodes, or concern for cancer (eg, coronary artery calcifications, thyroid nodules, radiographic emphysema)^a^ (n = 63)	
Letter only	8 (12.7)
Call only	7 (11.1)
Letter and call	27 (42.9)
Secure message	6 (9.5)
Free text entry: referral back to primary care for notification	22 (34.9)
For incidental findings (as described in the prior question) who is primarily/usually responsible for notifying the veteran at your site?^a^ (n = 63)	
LCS coordinator	14 (22.2)
Pulmonary	53 (84.1)
Primary care	29 (46.0)
Radiology	1 (1.6)

LCS = lung cancer screening; LDCT = low-dose CT; CBOC = community-based outpatient clinic; PCP = primary care provider; SDM = shared decision-making.

a Multiple selections allowed.

Respondents indicated perceived best ways to communicate a screen-detected pulmonary nodule finding. Most selected a phone call (81%) and a letter with educational material (73%), with just one-third indicating a visit with a provider was needed. Most did not think it is necessary to explain nodule characteristics (eg, shape, density) to patients beyond information in educational mailers (64%).

Current modes of result communication were analyzed across LCS program type and most included letters, phone calls, or both from either LCS coordinators or primary care providers (PCPs). When stratified by program type, letters from LCS coordinators were the most common modality of notification in centralized (93%) and hybrid (65%) program models, whereas letters from PCPs were the most common modality of notification in decentralized programs (80%).

Result communication varied across LungRADs category. Practices were similar in 89% of programs for LungRADS 1 and 2 results and relied on letters. LungRADS 3 results were primarily communicated via letter paired with telephone call (60%), letter only (21%), or call only (19%); this did not vary based on program model. The responsible provider calling with results was primarily the LCS coordinator in centralized (86%) and hybrid models (46%) and PCPs in decentralized models (60%). Similarly, LungRADS 4 result notification was done via letter and call (56%) or by telephone call only (44%) across all program models.

Regarding SIFs on LDCT scan not concerning for malignancy, most respondents (53 of 63, 84%) indicated that pulmonary providers were responsible for notifying patients. However, over one-third (29 of 63, 46%) also indicated that the responsibility for SIF management and communication was transitioned back to the PCP. Two-thirds (42 of 63, 66%) indicated that communication was done via letter, telephone call, or both.

## Discussion

Most LCS examinations with a nodule or incidental finding reported will require follow-up that should be communicated to the patient.^7^ To our knowledge, our study is the first to quantitatively evaluate the modes of communication used to deliver results to patients in a large integrated health system and has 2 important findings. First, regardless of LCS program model, most respondents think a telephone call is the best way to communicate findings of a pulmonary nodule; however, a call is only used with more high-risk findings. Second, incidental findings on LDCT scan are communicated through a variety of modalities, often relying on alerting PCPs to the results who then must decide on management and communication with the patient.

Overall, 81% of LCS coordinators thought a call was the best way to communicate the finding of a pulmonary nodule with patients. This form of communication is thought by both patients and clinicians to be of the highest quality.^2^ However, when further stratified by severity of findings, patients with normal or low risk pulmonary nodules (LungRADS 1 and 2) most often received a letter without a telephone call. These practices are in line with prior qualitative work.^8^ Like prior work,^8^ more concerning results (LungRADS 4) were communicated by call alone or in combination with a letter. It may be that time constraints and volume prevent a call to relay results of lower-risk examinations, indicating a need for LCS program administrative support to improve communication with patients, and additional collaboration with PCPs to avoid redundancy.

Management and communication of SIFs on LCS examinations can also impact patient care and distress. SIFs are common as in a retrospective review of 26,455 participants in the LDCT screened arm of the National Lung Screening Trial that found one-third had SIFs on at least 1 examination, with 89% considered reportable to the ordering provider.^7^ The most common were emphysema (43%) and coronary artery calcium (12%). Reporting of incidental findings varies widely among radiologists, with 1 study finding radiologists reported at least 1 incidental finding on 69% to 100% of studies, including it in the impression 0% to 75% of the time, applying the S modifier 0% to 51% of the time, and recommending additional testing in 34% to 69%.^9^ The lack of standardization is likely to pose a management dilemma as the uptake of LCS increases. In our study, respondents indicated that the LCS/pulmonary team was responsible for management of SIFs; however, management most often entailed transitioning responsibility for decision-making and communication with patients back to the PCP. This identifies a gap in care that would benefit from standardization and further evaluation to ensure patients with SIFs receive appropriate notification and evaluation to improve outcomes while minimizing the harms and costs of unnecessary downstream testing. It may be that expectation setting regarding mode for delivery of results, types of findings, and responsible provider could be included during SDM.

A variety of modalities are used to communicate LCS examination findings. Further work is needed to identify which of these represent best practices for communication between radiology, ordering provider, and patient to ensure optimal outcomes while minimizing potential harms.

## Financial/Nonfinancial Disclosures

None declared.
